# A 9‑gene expression signature to predict stage development in resectable stomach adenocarcinoma

**DOI:** 10.1186/s12876-022-02510-8

**Published:** 2022-10-14

**Authors:** Zining Liu, Hua Liu, Yinkui Wang, Ziyu Li

**Affiliations:** 1 Department of Gynecologic Oncology, National Cancer Center/National Clinical Research Center for Cancer/Cancer Hospital, Chinese Academy of Medical Sciences & Peking Union Medical College, Beijing, China; 2 Department of Gastrointestinal Oncology, Key Laboratory of Carcinogenesis and Translational Research (Ministry of Education), Peking University Cancer Hospital & Institute, Beijing, China; 3 Key laboratory of Carcinogenesis and Translational Research (Ministry of Education/Beijing), Gastrointestinal Cancer Center, Peking University Cancer Hospital & Institute, Beijing, 100142 China

**Keywords:** Gastric adenocarcinoma, Prognostic marker, Bioinformatics analysis, Tumor progression

## Abstract

**Background:**

Stomach adenocarcinoma (STAD) is a highly heterogeneous disease and is among the leading causes of cancer-related death worldwide. At present, TNM stage remains the most effective prognostic factor for STAD. Exploring the changes in gene expression levels associated with TNM stage development may help oncologists to better understand the commonalities in the progression of STAD and may provide a new way of identifying early-stage STAD so that optimal treatment approaches can be provided.

**Methods:**

The RNA profile retrieving strategy was utilized and RNA expression profiling was performed using two large STAD microarray databases (GSE62254, n = 300; GSE15459, n = 192) from the Gene Expression Omnibus (GEO) and the RNA-seq database within the Cancer Genome Atlas (TCGA, n = 375). All sample expression information was obtained from STAD tissues after radical resection. After excluding data with insufficient staging information and lymph node number, samples were grouped into earlier-stage and later-stage. Samples in GSE62254 were randomly divided into a training group (n = 172) and a validation group (n = 86). Differentially expressed genes (DEGs) were selected based on the expression of mRNAs in the training group and the TCGA group (n = 156), and hub genes were further screened by least absolute shrinkage and selection operator (LASSO) logistic regression. Receiver operating characteristic (ROC) curves were used to evaluate the performance of the hub genes in distinguishing STAD stage in the validation group and the GSE15459 dataset. Univariate and multivariate Cox regressions were performed sequentially.

**Results:**

22 DEGs were commonly upregulated (n = 19) or downregulated (n = 3) in the training and TCGA datasets. Nine genes, including MYOCD, GHRL, SCRG1, TYRP1, LYPD6B, THBS4, TNFRSF17, SERPINB2, and NEBL were identified as hub genes by LASSO-logistic regression. The model achieved discrimination in the validation group (AUC = 0.704), training-validation group (AUC = 0.743), and GSE15459 dataset (AUC = 0.658), respectively. Gene Set Enrichment Analysis (GSEA) was used to identify the potential stage-development pathways, including the PI3K-Akt and Calcium signaling pathways. Univariate Cox regression indicated that the nine-gene score was a significant risk factor for overall survival (HR = 1.28, 95% CI 1.08–1.50, *P* = 0.003). In the multivariate Cox regression, only SCRG1 was an independent prognostic predictor of overall survival after backward stepwise elimination (HR = 1.21, 95% CI 1.11–1.32, *P* < 0.001).

**Conclusion:**

Through a series of bioinformatics and validation processes, a nine-gene signature that can distinguish STAD stage was identified. This gene signature has potential clinical application and may provide a novel approach to understanding the progression of STAD.

**Supplementary Information:**

The online version contains supplementary material available at 10.1186/s12876-022-02510-8.

## Introduction

Stomach adenocarcinoma (STAD) is the fifth most frequently diagnosed cancer and the fourth-leading cause of cancer-related death worldwide [1]. The long-term prognosis of patients with STAD differs significantly as a function of tumor stage as assessed by the 8th American Joint Committee on Cancer (AJCC) tumor, node, metastasis (TNM) system. At present, although surgical resection is the only possible curative treatment for resectable STAD in stages I to III, a satisfactory result is only achieved in early-stage STAD cases. According to the SEER database, the 10-year survival rate for patients below stage IIa is approximately 70% but for those above stage IIb, it is only about 50% [2]. Preoperative treatment is particularly important for patients with mid-to-late stage STAD and has been recommended in various guidelines for many years [3, 4,5]

To identify whether drug or surgical treatment should be performed in the first instance, an accurate preoperative staging method for STAD is imperative. Microarray technology and high-throughput transcriptome profiling have provided new insights into tumor occurrence and development. It may be possible to link the gene expression profile of STAD with certain phenotypes or clinical features. As such, a set of gene signatures could potentially be used to profile STAD at different stages, further assisting clinicians in treatment decision-making in order to achieve optimal outcomes for STAD patients. Current radiological measures, including widely-applied computed tomography (CT), have only limited accuracy, especially in lymph node assessment [6]. Considerable under-staging still occurs.

More importantly, as TNM staging is still the most accurate indicator of STAD patient prognosis, there is an urgent need to identify the relationships between changes in gene expression and disease stage progression. This could assist oncologists to identify commonalities in tumorigenesis and development among this highly heterogeneous cancer type. Previous studies have focused on the direct links between gene expression and survival using open-access data [7–9]. However, it is clear that a patient’s duration of survival partially depends on the treatment they receive: the resection type (D2 or not) they received, their compliance with postoperative chemotherapy, and their choices for second-line treatment upon relapse. The TNM stage may be a more direct characteristic that reflects the mechanism of ontogenesis in some ways. To date, few studies have focused on TNM staging and this may be due to differences in the staging criteria applied to previous public data, which hampers the ability of researchers to link genes and staging data. Therefore, unified staging criteria based on the latest 8th AJCC edition are required.

The present study aimed to screen gene expression signatures for the discrimination of earlier and later TNM stages in local, non-metastatic STAD patients using systematic bioinformatic analysis of transcriptomic data.

## Methods

### Data sources and data pre-processing

#### TCGA dataset

The RNA sequencing data for STAD tissues were downloaded from the TCGA dataset (https://tcga-data.nci.nih.gov/tcga/) and contained 375 STAD samples with complete clinical and pathological information. The messenger RNA (mRNA) expression dataset was then extracted. Samples were excluded if: (1) the data were missing T stage information, (2) less than 16 lymph nodes were retrieved, (3) the patient had distant metastasis (M1), or (4) the patient had received preoperative treatment. In total, 162 eligible samples were screened from the 374 samples. The T and N stages and overall TNM stage were modified according to the latest AJCC 8th edition criteria (Additional file 1: Table S1). Patients who were classified as 8th edition TNM stages I to IIa were combined into an earlier-stage group (I-IIa) and those classified as stages Iib to III were combined into a later-stage group (Iib-III). To assure the accuracy of the results, features with less than two counts in more than 50% of the samples were discarded.

#### Training-validation dataset

The GEO website includes five publicly available series that contain more than 30 STAD tissue samples with complete TNM stage information (GSE15459, GSE26942, GSE62254, GSE29272, and GSE27342). None of these were staged according to the AJCC 8th edition. Only one publicly available gene expression profile (GSE62254) has detailed information on the pathological T stage, the number of retrieved and positive lymph nodes, and metastasis. For this reason, GSE62254 was selected as the training-validation dataset. The expression data of the 300 STAD samples in GSE62254 were generated using the GPL570 platform (Affymetrix Human Genome U133 plus 2.0 Array) and downloaded from the GEO database (http://www.ncbi.nlm.nih.gov/geo/). For microarray datasets, ineligible records were excluded according to the same principles as described above: (1) missing T stage information, (2) less than 16 retrieved lymph nodes, and (3) distant metastasis (M1). In total, 262 samples met these criteria.

#### Validation set 2

To verify the robust performance of the model fitting, GSE15459—obtained from the same GPL570 platform—was adopted as the second validation set. GSE15459 contains 192 qualified genome-wide mRNA expression profiles of primary STAD patients. The staging system in GSE15459 is based on the AJCC 6th edition TNM system, ranging from I to IV. As this database lacks clinical data on the number of retrieved/positive lymph nodes and the metastasis status, the GSE15459 data could not be transformed into the AJCC 8th staging system. Therefore, the stage I samples were classified as the earlier-stage group (N = 31) and the stages II to IV samples (N = 161) were classified as the later-stage group. Despite the diagnostic accuracy and criteria divergence, the diagnostic scope of stage I in the 6th edition is similar to that of stage I-IIA in the 8th edition (except for T2N1). Thus, agreement under the same prediction model was expected (Table Additional file 1: S1).

#### Outlier detection and removal

The TCGA dataset (N = 159) and GSE62254 dataset (N = 262) were separately subjected to outlier analysis using hierarchical cluster analysis via the “hclust” function in the WGCNA package [10]. After outlier removal, expression data were obtained from 156 subjects in the TCGA dataset (44 in the earlier-stage group and 112 in the later-stage group) and 258 subjects in the training-validation microarray dataset (73 in the earlier-stage group and 185 in the later-stage group; Additional file 5: Figure S1A, S1B).

#### Data splitting

The training-validation set was further divided into a training set (66.7%) and a validation set (33.3%) at a 2:1 ratio. A stratified sampling method was adopted according to grouping (earlier-stage vs. later-stage) using the function “strata” in the “sampling” R package. After sampling, there were 49 earlier-stage and 123 later-stage subjects in the training set and 24 earlier-stage and 62 later-stage subjects in validation set 1.

### Selection of differentially expressed genes (DEGs)

Differentially expressed genes were identified using the LIMMA package (version 3.42.2) for microarray data and DESeq2 (version 1.26.0) for RNA-seq data in R 3.6.2 [11, 12]. Significant DEGs were detected according to the following criteria: (1) absolute fold-change > 1.5, (2) normalized (NOM) *P* value < 0.05, and (3) q-value (false discovery rate [FDR]) < 0.25. Overlapping DEGs between the GEO and TCGA database were reserved for subsequent study. Heat maps and volcano plots of the DEGs were drawn using the “ggplots” and “pheatmap” packages in R.

### Enrichment analysis of DEGs

Functional enrichment analysis included Gene Ontology (GO) analysis and Kyoto Encyclopedia of Genes and Genomes (KEGG) analysis. GO and KEGG analyses were carried out using “clusterProfiler” in R (version 3.14.3) [13–15]. GO analysis encompassed biological processes, cellular components, and molecular functions. Gene Set Enrichment Analysis (GSEA) was also performed using the “gsekegg” function with 1,000 permutations of the gene sets and a log2 ratio of classes as the metric for ranking genes. For both enrichment analysis and GSEA, pathways with both a NOM *P*-value < 0.05 and FDR < 0.25 were considered significant, as recommended previously [16]. Additionally, only those pathways with an absolute normalized enrichment score (NES) > 1 were adopted in the GSEA results.

### Establishment of outcome signature with LASSO logistic regression model

The Least Absolute Shrinkage and Selection Operator (LASSO) method was applied to reduce the dimensions of the data and select the DEGs that best distinguished the data. This was achieved using the “glmnet” (version 4.0-2) package in the training microarray data. In the LASSO model, the minimum criterion (λ) based on 10-fold cross-validations was chosen. A multivariate logistic regression model was used to build a model for predicting later-stage cancer. The predictive index of each sample was calculated according to the constructed prognostic signatures based on the following formula: prediction index = ${\sum }_{i=1}^{n}{\upbeta }\text{i} \times \text{X}\text{i}$, where βi represents the coefficient obtained from LASSO-logistic regression and Xi indicates the relative expression level of each selected gene. The area under the curve (AUC) was calculated in the training, validation 1, and validation 2 datasets using the “rms” package.

### Statistical analysis

All data were analyzed using R (version 3.6.2). Comparisons between the two groups were made using the χ^2^ test (nominal data), Wilcoxon rank test (nonparametric continuous data), or Student’s t-test (Gaussian continuous data), as appropriate. For predictive ability, the AUC was required to be equal to or higher than 0.65 with a 95% confidence interval (95% CI) excluding 0.5; an AUC ≥ 0.7 was considered to reflect good prediction or discrimination. We also compared the predictive ability of our gene signature with previously published prognostic signatures [17–25]. The Venkatraman permutation test was used to compare the paired ROC curves based on different signatures [26]. The prognosis values of the hub genes with the same probe IDs were inspected using Kaplan-Meier analysis based on the log-rank test. The relationships between clinicopathological factors and both long-term overall survival (OS) and disease-free survival (DFS) were assessed using univariate Cox regression analysis. Covariates that achieved a *P*-value < 0.05 in the univariate analyses were included in the multivariate analysis. A backward stepwise approach was used to identify possible predictors of OS among the candidate variables. The AIC was used to set a limit on the total number of variables included in the final model. *P*-values < 0.05 were considered statistically significant. The “sva” package in R was used to remove the batch effect between the datasets using the same platform, if necessary [27].

## Results

### Identification of DEGs

**Fig. 1:**
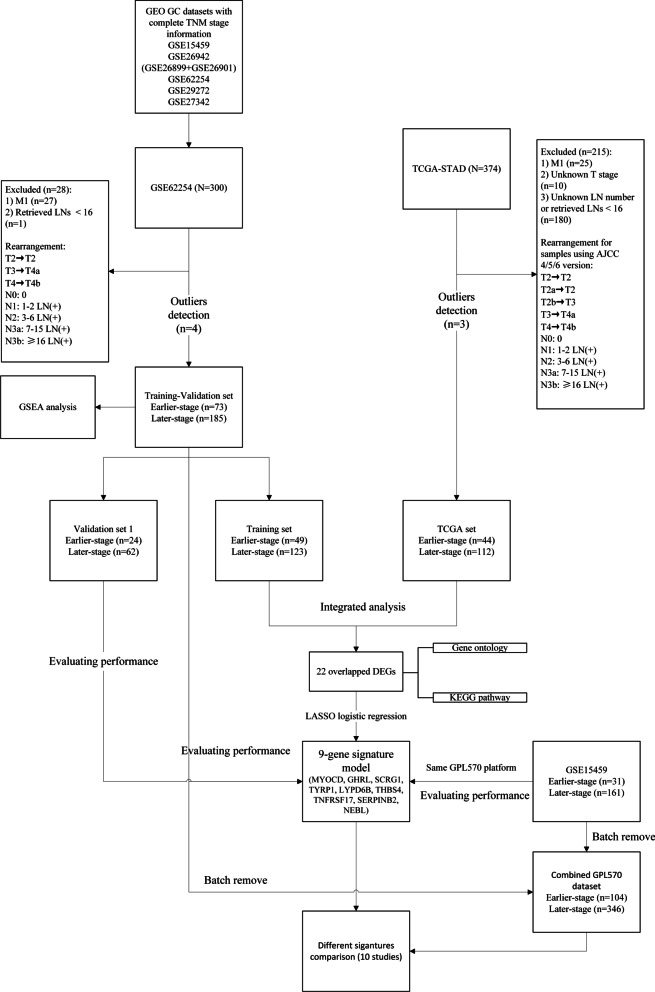
Flow chart of samples selection

A detailed flow chart of the prognostic predictive model in this study is shown in Fig. 1. The detailed clinical features of the TCGA, training-validation, and validation 2 datasets before outlier removal are shown in Additional file 2: Table S2.

**Fig. 2:**
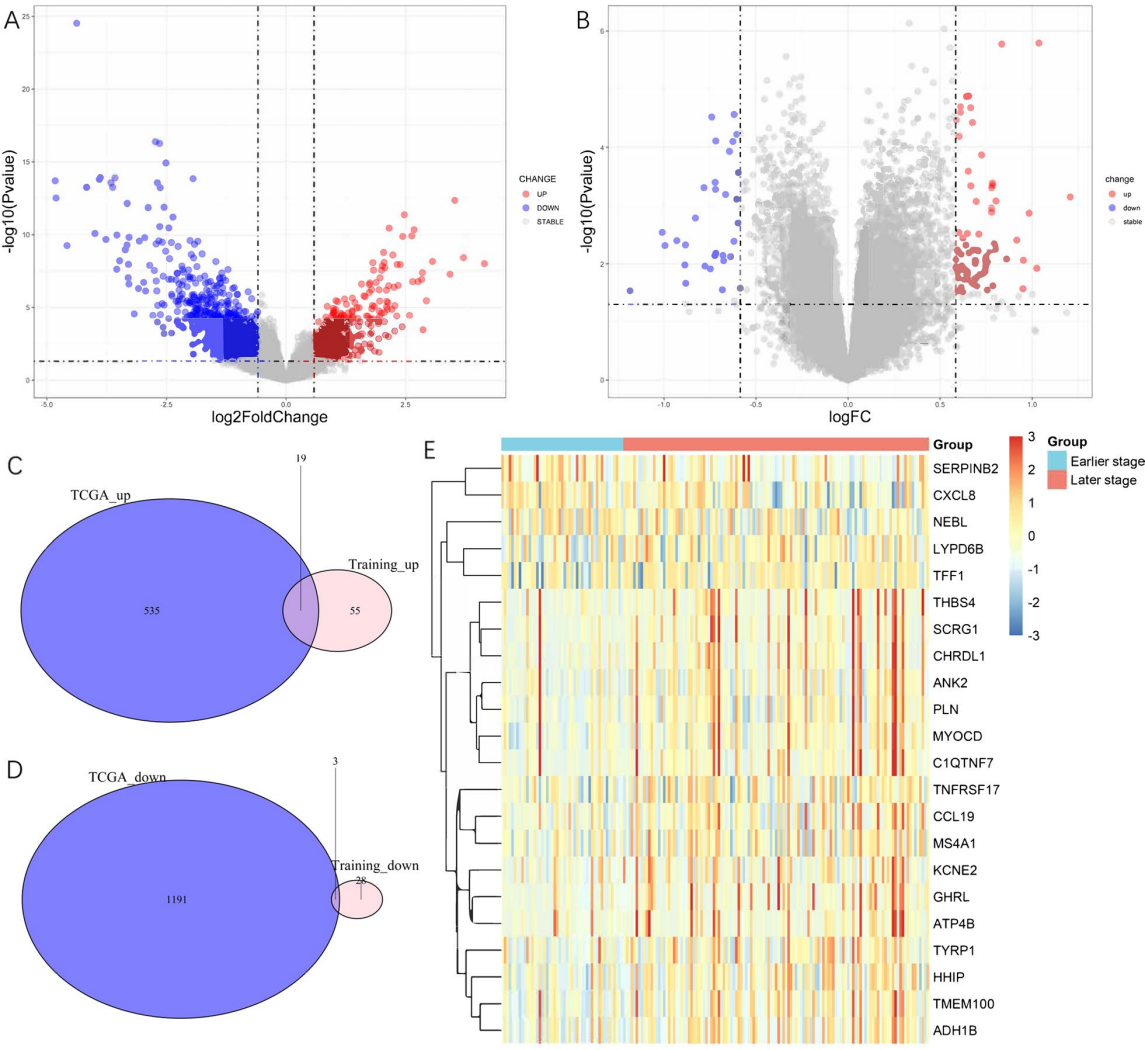
Differentially expressed stage-related genes for STAD in (**A**) TCGA and (**B**) training datasets; Venn diagram showing overlapped (**C**) upregulated and **D** down-regulated DEGs between TCGA and training dataset; **E** the expression heatmap of the 22 overlapped DEGs

The DEGs between the earlier-stage and later-stage samples in the TCGA dataset and training set were screened. Detailed patient information from both databases is shown in Tables 1 and 2. Compared to the earlier-stage tumors, a total of 1748 DEGs, including 554 upregulated genes and 1194 downregulated genes, were identified in the later-stage group of the TCGA dataset (Fig. 2A) while 74 upregulated genes and 31 downregulated DEGs between the later-stage and earlier-stage samples were identified in the training set (Fig. 2B). Among the two datasets, 22 overlapping DEGs (19 upregulated and 3 downregulated) were identified (Fig. 2C, D). All DEGs are listed in Additional file 3: Table S3. Heatmap analysis was used to determine the relative expression levels of these 22 DEGs in the different groups (Fig. 2E).

**Table 1:** Demographic and clinicopathologic characteristics in training and validation cohorts (GSE62254)

Variables	Training	Validation	*P* value
N	172	86	
Sex			
Female	61 (35.5)	24 (27.9)	0.281
Male	111 (64.5)	62 (72.1)	
Age (years)			
≤ 65	105 (61.0)	43 (50.0)	0.119
> 65	67 (39.0)	43 (50.0)	
Signet ring			
No	144 (83.7)	77 (89.5)	0.286
Yes	28 (16.3)	9 (10.5)	
Perineural invasion			
No	87 (63.0)	51 (68.9)	0.481
Yes	51 (37.0)	23 (31.1)	
Lymphovascular invasion			
No	43 (27.2)	21 (25.9)	0.953
Yes	115 (72.8)	60 (74.1)	
T stage			
2–3	113 (65.7)	57 (66.3)	0.817
4a	50 (29.1)	26 (30.2)	
4b	9 ( 5.2)	3 ( 3.5)	
N stage			
0	19 (11.0)	13 (15.1)	0.755
1	37 (21.5)	16 (18.6)	
2	45 (26.2)	22 (25.6)	
3a	42 (24.4)	24 (27.9)	
3b	29 (16.9)	11 (12.8)	
Number of positive lymph nodes	8.06 ± 9.27	7.87 ± 9.96	0.879
Lauren			
diffuse	81 (47.1)	30 (34.9)	0.126
intestinal	79 (45.9)	51 (59.3)	
mixed	12 ( 7.0)	5 ( 5.8)	
Tumor location (%)			
Cardia/Upper	19 (11.0)	7 ( 8.1)	0.613
Middle	65 (37.8)	30 (34.9)	
Antrum/Distal	88 (51.2)	49 (57.0)	
ACRG.sub (%)			
EMT	25 (14.5)	10 (11.6)	0.809
MSI	41 (23.8)	24 (27.9)	
TP53neg	59 (34.3)	31 (36.0)	
TP53positive	47 (27.3)	21 (24.4)	
Stage by AJCC 8th			
Earlier stage (≤ IIa)	49 (28.5)	24 (27.9)	1.000
Later stage (> IIa)	123 (71.5)	62 (72.1)

**Table 2:** Demographic and clinicopathologic characteristics in TCGA dataset

Variables	Overall
N	156
Sex	
Female	43 (27.6)
Male	113 (72.4)
Age (years)	
≤ 65	52 (33.8)
> 65	102 (66.2)
Signet ring	
No	150 (96.2)
Yes	6 ( 3.8)
T stage	
1	14 ( 9.0)
2	36 (23.1)
3	52 (33.3)
4a	39 (25.0)
4b	15 ( 9.6)
N stage	
0	44 (28.2)
1	27 (17.3)
2	33 (21.2)
3a	31 (19.9)
3b	21 (13.5)
Lauren	
Diffuse	31 (19.9)
Intestinal	93 (59.6)
Not specified	32 (20.5)
Number of positive lymph nodes	7.15 ± 10.11
Stage by AJCC 8th	
Earlier stage (≤ IIa)	44 (28.2)
Later stage (> IIa)	112 (71.8)

**Fig. 3:**
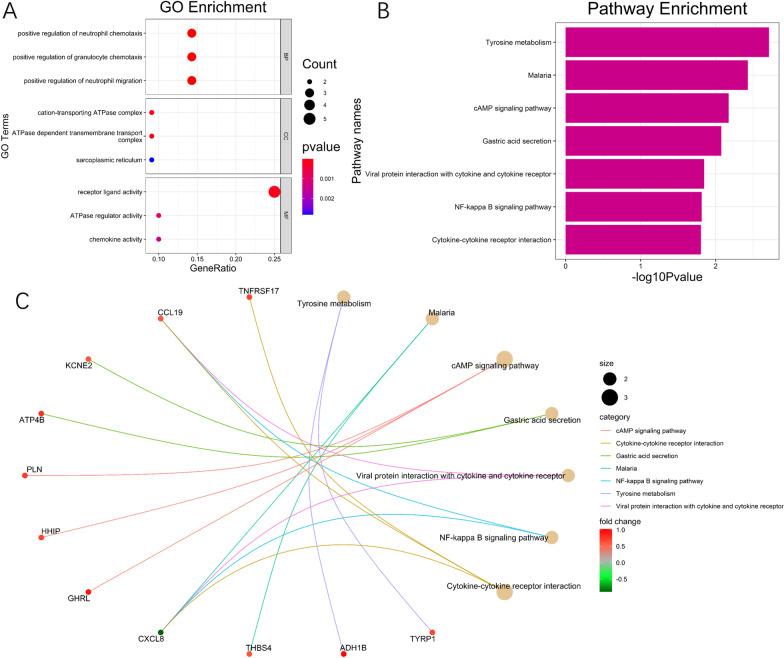
**A** GO analysis of the 22 overlapped DEGs; **B** KEGG pathway of DEGs. **C** Net plot of the pathways enriched with DEGs, as identified by KEGG pathway analysis

All overlapping DEGs were submitted to GO and KEGG pathway analyses. The top three GO enrichment terms for target genes in the biological processes of ontology, cellular components of ontology, and molecular function of ontology are shown separately in Fig. 3A; all seven enriched KEGG terms are presented in Fig. 3B. The results showed that “positive regulation of cytosolic calcium ion concentration” and “calcium ion transport into cytosol” were the most enriched GO terms, while “tyrosine metabolism”, “malaria”, and “cAMP signaling pathway” were the most enriched KEGG terms. The DEGs and their interactions with KEGG pathways are visualized in Fig. 3C.

### Predicting pathological stage with binomial LASSO logistic regression

**Fig. 4:**
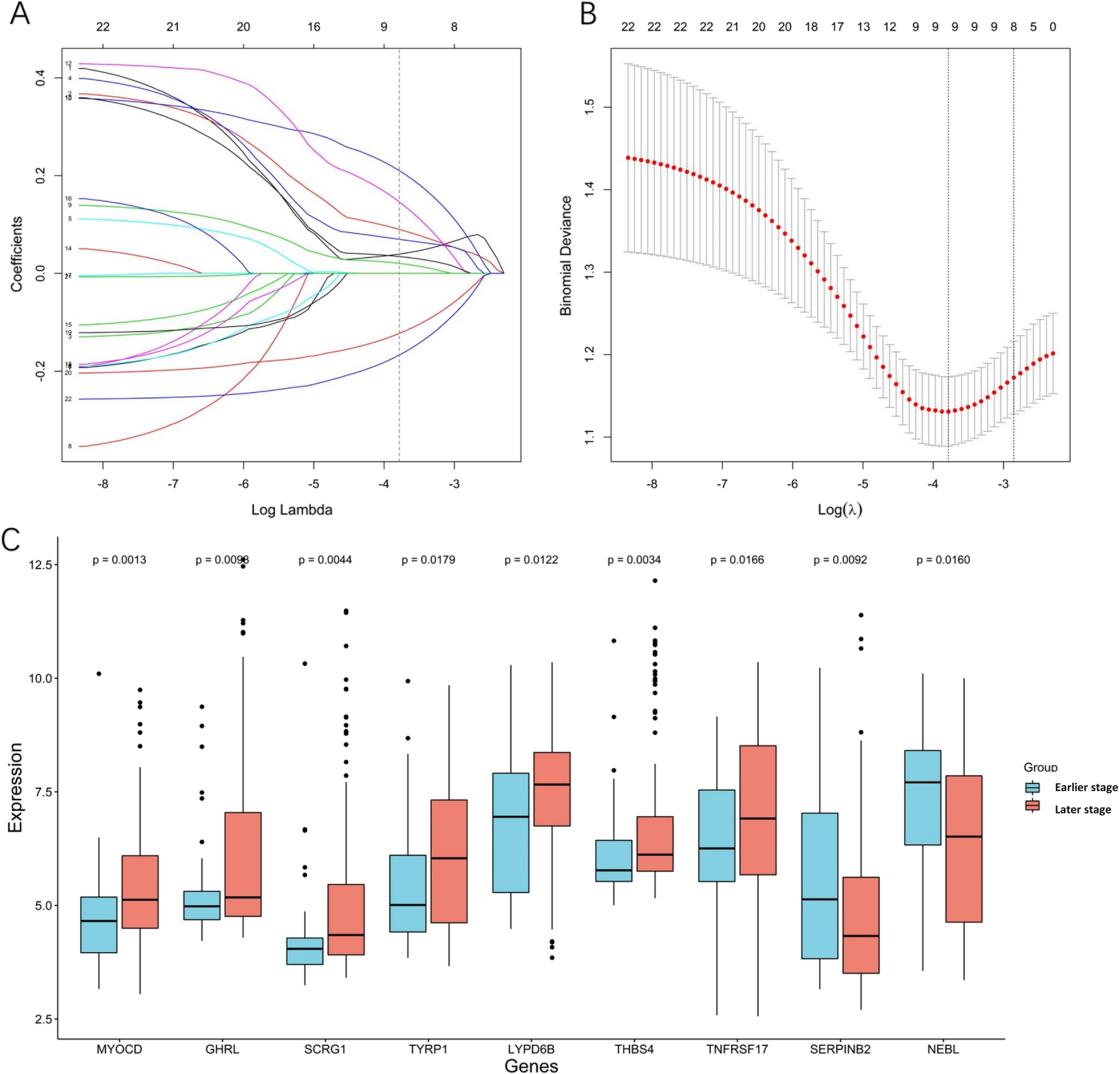
LASSO logistic analysis via 10‑fold cross‑validation with minimum criteria. **A** Tuning parameter selection via 10‑fold cross‑validation with minimum criteria in the LASSO model. **B** LASSO coefficient profiles of 22 candidate DEGs. LASSO. **C** The expression level of the nine hub genes between the earlier-stage and later-stage groups as identified by LASSO regression

To examine the DEGs with the best discriminative ability for stage prediction, and to minimize multicollinearity, LASSO logistic regression was employed. Feature selection was performed based on the training dataset with the 22 identified DEGs. LASSO regression yielded a model with nine predictors (seven upregulated and two downregulated) that minimized binomial deviance and enhanced sparsity (Fig. 4A, B). These nine hub genes showed significant upregulation/downregulation between the two stage groups (Fig. 4C). The Kaplan-Meier plots indicated that overexpression of MYOCD, SCRG1, TYRP1, and THBS4 was associated with significantly poorer survival, while upregulation of GHRL and LYPD6B and downregulation of SERINB2 and NEBL tended to be associated with poorer survival. Only TNFRSF17 showed no expression-related survival trend (Additional file 6: Figure S2A–I).

**Fig. 5:**
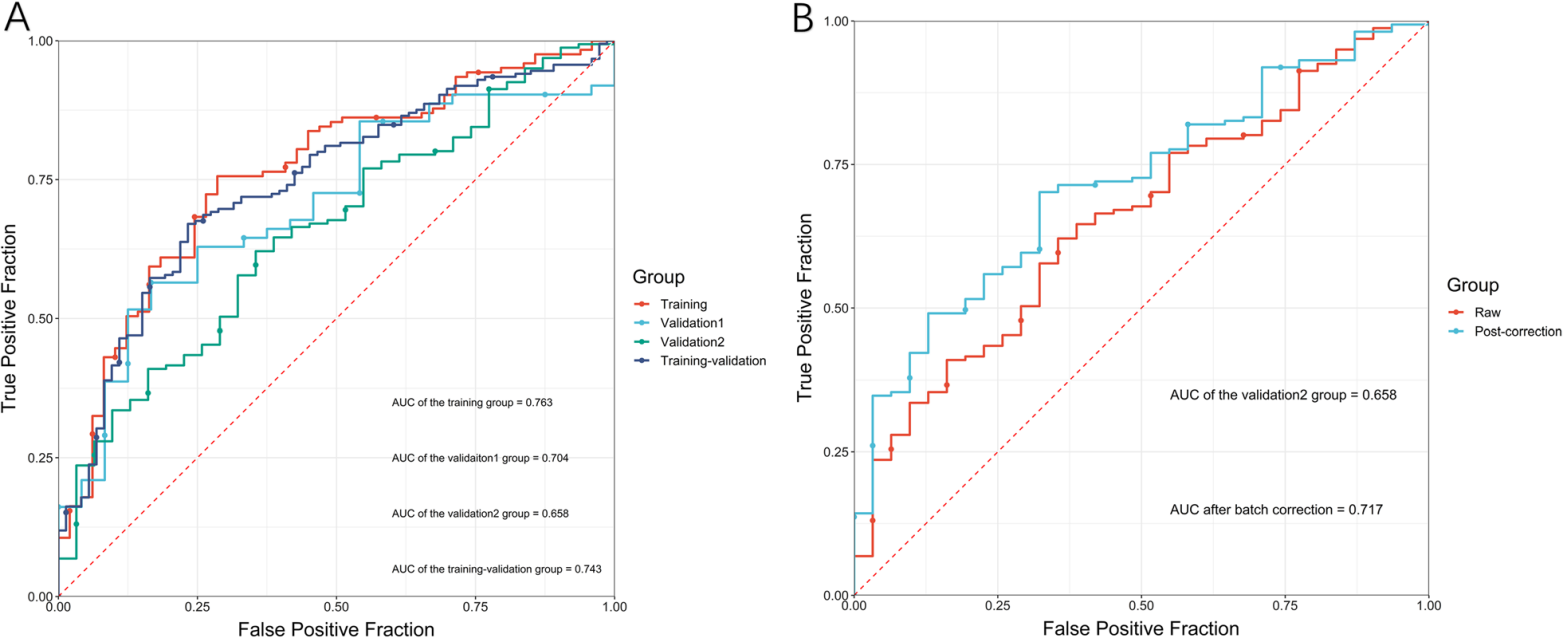
Receiver operating characteristic curve based on the training, validation set 1, training-validation and validation set 2 (GSE15459); The ROC performances in validation set 2 before and after batch correction

The nine hub genes (MYOCD, GHRL, SCRG1, TYRP1, LYPD6B, THBS4, TNFRSF17, SERPINB2, and NEBL) were included in a multivariate logistic regression model. The obtained coefficients of each identified DEG were then used to form the nine-gene model (Table 3). No reverse sign was observed in any of the covariates within the univariate and multivariate regressions. The ability of the nine-gene signature to predict TNM stage was evaluated by ROC curves and AUC analysis. In the training set, the AUC was 0.763 (0.685–0.841). The prediction model also achieved satisfactory performance with an AUC of 0.704 (0.587–0.821) in validation set 1 and an AUC of 0.743 (0.679–0.808) in the merged training-validation set. The prediction model performed moderately in validation set 2 with an AUC of 0.658 (0.558–0.758). The AUCs in each data set are presented in Fig. 5A.

**Table 3:** LASSO regression results. Genes selected by the LASSO logistic regression, with the estimated coefficients and odds ratio

Gene	Coefficient	Odds ratio
MYOCD	0.02258333	1.0228403
GHRL	0.14381831	1.1546743
SCRG1	0.10258011	1.1080261
TYRP1	0.03295502	1.0335041
LYPD6B	0.30825199	1.3610439
THBS4	0.27385904	1.3150294
TNFRSF17	0.04663749	1.0477421
SERPINB2	−0.18898024	0.8278029
NEBL	−0.24998835	0.7788099

A significant batch effect between GSE15459 (validation set 2) and GSE62254 (training-validation set) was observed. Because the two series used the same GPL570 platform, batch correction for validation set 2 with reference to the training-validation set was then performed. Boxplots of the merged dataset before and after batch effect removal are presented in Additional file 7: Figures S3A and S3B, respectively. There was an obvious improvement in the AUC value, which increased to 0.717 (0.627–0.806) after batch correction (Fig. 5B).

The nine-gene model was then applied to several clinical phenotypes. The prediction model performed well in forecasting lymph node metastasis (AUC: 0.728, 95% CI 0.647–0.808), signet ring (AUC: 0.711, 95% CI 0.617–0.805), and Lauren diffuse type (AUC: 0.707, 95% CI 0.643–0.771) STAD. The model achieved a moderate predictive value for T4 tumors (Table 4).

**Table 4:** The AUC performances of the 9 hub genes on other clinicopathologic phenotypes

Variables	AUC (95% CI)
N+	0.728 (0.647–0.808)
T4	0.687 (0.617–0.756)
Signet ring	0.711 (0.617–0.805)
Lauren diffuse type	0.707 (0.643–0.771)
Diffuse + Mixed	0.709 (0.647–0.772)
Antrum	0.602 (0.531–0.673)
Cardia	0.563 (0.441–0.685)
Age (> 65 years)	0.611 (0.543–0.680)
Gender (male)	0.608 (0.533–0.683)

### Identification of KEGG pathways related to the TNM stage using GSEA

**Fig. 6:**
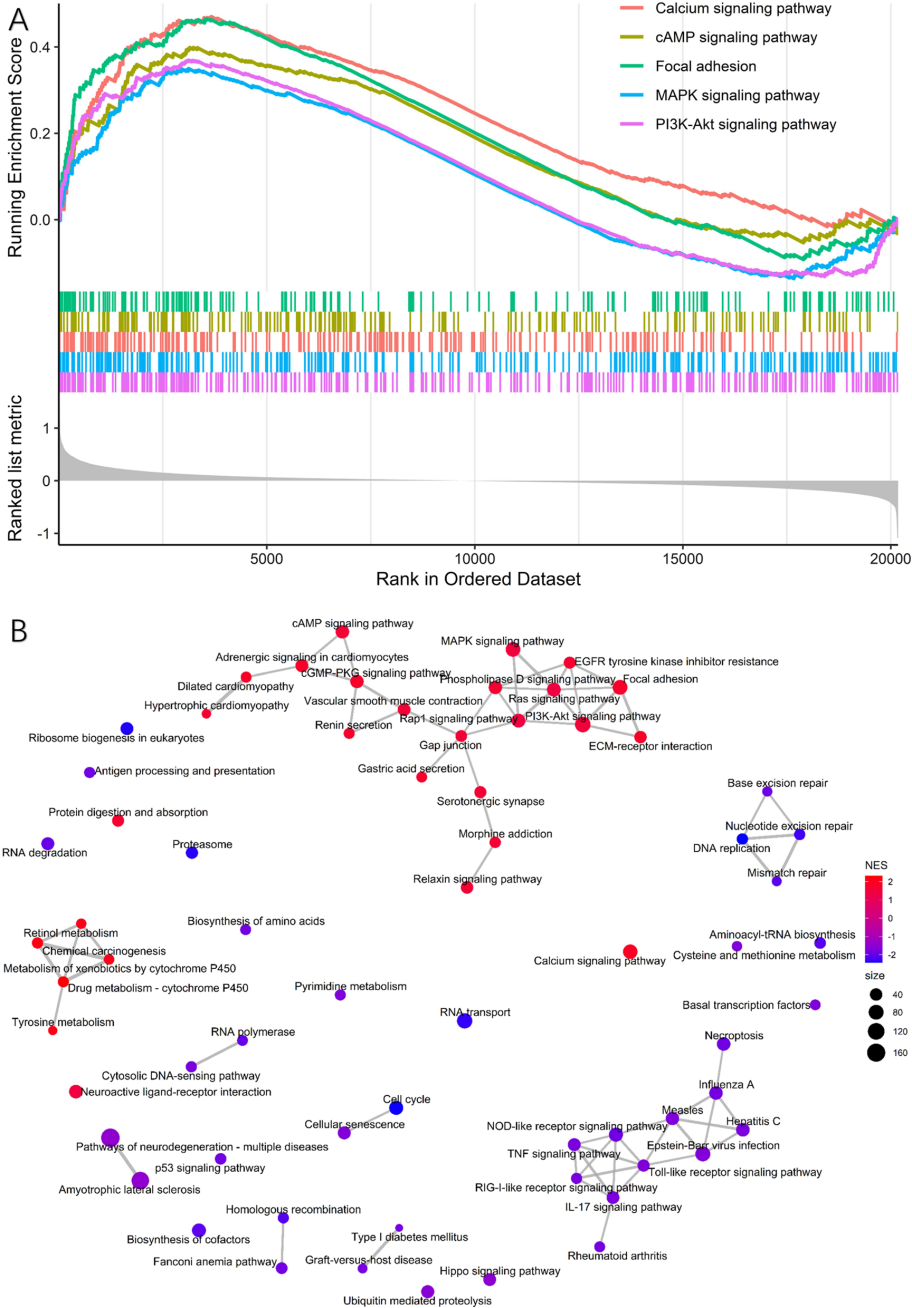
Gene set enrichment analysis analysis based on the training-validation set **A** Top five GSEA enrichment analysis results of the KEGG pathways for the later-stage group. **B** Network plots for GSEA. Network plot showing enriched upregulated pathways (in red) and downregulated pathways (in blue) for gene expression data samples with higher stage. Top 50% significant KEGG were included in this network

To improve our understanding of the gene expression changes that accompany stage development, GSEA was performed using the training-validation set (GSE62254). From this, 134 (62 upregulated and 72 downregulated) significantly enriched pathways were identified (*P* < 0.05, FDR < 0.25). All of the top 10 significantly enriched pathways were upregulated (Fig. 6A). The “PI3K-Akt signaling pathway” was the most significantly upregulated, followed by the “MAPK signaling pathway”, “Calcium signaling pathway”, “cAMP pathway”, and “focal adhesion”. A network of gene sets in the first half (N = 67) was constructed to illustrate the pathway interactions (Fig. 6B). The details of the significantly enriched gene sets are provided in Additional file 4: Table S4.

### Exploring the prognostic significance of the nine genes and other clinicopathological factors

We further investigated the prognostic impact of the nine selected genes together with various clinicopathologic and genomic features. As the ACRG cohort had the most sophisticated clinical information and molecular subtypes, both the training-validation dataset (N = 258) and the original dataset (N = 300) were used to achieve robust results. Univariate Cox analysis revealed that higher signature score, tumor location, total resection, T stage, N stage, MLH1 positivity, diffuse Lauren type, poor differentiation, ACRG subtype (especially EMT), absent chemotherapy, mesenchymal phenotype, and Borrmann type IV were risk factors for OS and/or DFS either in the training-validation dataset (Table 5A) or in the complete ACRG cohort (Table 5B). Specifically, four of the nine selected genes, i.e., MYOCD, SCRG1, TYRP1, and THBS4, were significantly correlated with survival as continuous variables. All statistically significant variables were then included in a multivariate Cox regression using the backward stepwise algorithm for covariate selection. The results showed that N stage, chemotherapy, and SCRG1 expression level (training-validation dataset: HR 1.21, 95% CI 1.11–1.32, *P* < 0.001; ACRG cohort: 1.14, 95% CI 1.05–1.24, *P* = 0.001) were significant covariates in both datasets (Table 6A, B), while T stage and MLH1 status were significant covariates only in the complete ACRG cohort (Table 6B). Other features, e.g., ACRG subtype, mesenchymal phenotype, and other selected genes, were ruled out in both datasets using the same algorithms.

**Table 5:** (A) Univariate Cox regression in Training-Validation dataset (N = 258). (B) Univariate Cox regression in the whole ACRG cohort (N = 300)

Variables	Overall survival		Disease free survival	
	Hazard ratio	*P* value	Hazard ratio	*P* value
(A) N = 258				
Male	0.98 (0.67–1.45)	0.940	1.01 (0.66–1.54)	0.977
Age (per 1 year increase)	1.01 (0.99–1.03)	0.255	1.00 (0.98–1.02)	0.893
Tumor location				
Upper	1.00	1.000	1.00	1.000
Middle	0.61 (0.34–1.08)	0.089	0.76 (0.40–1.44)	0.397
Lower	0.56 (0.32–0.99)	0.045	0.56 (0.30–1.07)	0.082
Whole	2.91 (0.66–12.82)	0.158	3.33 (0.74–15.02)	0.117
Total resection	0.34 (0.23–0.50)	< 0.001	0.31 (0.21–0.48)	< 0.001
T stage				
T2-3	1.00	1.000	1.00	1.000
T4a	2.48 (1.70–3.61)	< 0.001	2.73 (1.81–4.13)	< 0.001
T4b	1.73 (0.79–3.78)	0.169	1.69 (0.72–3.94)	0.229
N stage				
N0	1.00	1.000	1.00	1.000
N1	2.79 (1.06–7.37)	0.039	2.73 (0.92–8.13)	0.070
N2	2.08 (0.79–5.54)	0.140	1.71 (0.56–5.20)	0.344
N3a	4.82 (1.90–12.25)	0.001	5.60 (1.98–15.83)	0.001
N3b	10.09 (3.91–26.03)	< 0.001	11.81 (4.13–33.77)	< 0.001
T4 stage	2.35 (1.64–3.38)	< 0.001	2.54 (1.71–3.79)	< 0.001
N + stage	3.87 (1.58–9.48)	0.003	4.04 (1.48–10.99)	0.006
High stage	2.18 (1.36–3.50)	0.001	2.35 (1.37–4.02)	0.002
MLH1 positivity	1.76 (1.09–2.85)	0.021	1.86 (1.07–3.23)	0.027
Lauren classification				
Intestinal	1.00	1.000	1.00	1.000
Mixed	2.18 (1.13–4.19)	0.020	1.89 (0.85–4.23)	0.120
Diffused	1.59 (1.09–2.33)	0.017	1.49 (0.98–2.25)	0.062
Poor differentiation	1.50 (1.03–2.17)	0.035	1.40 (0.93–2.11)	0.106
ACRG subtype				
TP53 negative	1.00	1.000	1.00	1.000
TP53positive	0.85 (0.53–1.36)	0.496	0.97 (0.58–1.63)	0.904
MSI	0.65 (0.39–1.09)	0.107	0.63 (0.34–1.16)	0.134
EMT	1.86 (1.14–3.06)	0.014	2.08 (1.23–3.51)	0.007
Chemotherapy	0.55 (0.35–0.85)	0.007	0.55 (0.34–0.88)	0.012
Mesenchymal phenotype	1.93 (1.32–2.81)	0.001	2.09 (1.38–3.15)	< 0.001
9-gene score	1.28 (1.08–1.50)	0.003	1.30 (1.10–1.55)	0.003
MYOCD	1.38 (1.23–1.55)	< 0.001	1.42 (1.25–1.61)	< 0.001
GHRL	1.03 (0.93–1.14)	0.585	1.04 (0.94–1.16)	0.399
SCRG1	1.27 (1.18–1.38)	< 0.001	1.30 (1.19–1.41)	< 0.001
TYRP1	1.20 (1.08–1.33)	0.001	1.22 (1.09–1.36)	0.001
LYPD6B	1.00 (0.89–1.13)	0.982	0.96 (0.85–1.09)	0.557
THBS4	1.27 (1.15–1.40)	< 0.001	1.29 (1.16–1.44)	< 0.001
TNFRSF17	0.95 (0.85–1.05)	0.298	0.95 (0.85–1.06)	0.388
SERPINB2	1.00 (0.90–1.10)	0.955	0.94 (0.84–1.06)	0.34
NEBL	1.01 (0.92–1.11)	0.865	1.01 (0.91–1.12)	0.854
Borrmann type				
Borrmann I or EGC	1.00	1.000	1.00	1.000
Borrmann II	0.71 (0.29–1.73)	0.455	0.62 (0.25–1.54)	0.302
Borrmann III	1.49 (0.64–3.44)	0.352	1.24 (0.53–2.90)	0.615
Borrmann IV	3.62 (1.48–8.89)	0.005	3.31 (1.33–8.25)	0.010
(B) N = 300				
Male	0.90 (0.65–1.27)	0.559	0.96 (0.66–1.39)	0.825
Age (per 1 year increase)	1.01 (1.00–1.03)	0.181	1.00 (0.99–1.02)	0.715
Tumor location				
Upper	1.00	1.000	1.00	1.000
Middle	1.09 (0.76–1.56)	0.631	1.21 (0.82–1.78)	0.330
Lower	1.66 (1.02–2.70)	0.041	1.62 (0.93–2.83)	0.087
Whole	3.27 (1.42–7.56)	0.006	2.21 (0.80–6.11)	0.127
Subtotal resection	0.38 (0.27–0.52)	< 0.001	0.38 (0.27–0.55)	< 0.001
T stage				
T2-3	1.00	1.000	1.00	1.000
T4a	2.37 (1.69–3.32)	< 0.001	2.52 (1.73–3.67)	< 0.001
T4b	2.51 (1.46–4.32)	< 0.001	2.71 (1.53–4.79)	0.001
N stage				
N0	1.00	1.000	1.00	1.000
N1	1.74 (0.86–3.54)	0.124	1.71 (0.76–3.83)	0.191
N2	3.37 (1.65–6.85)	< 0.001	3.94 (1.77–8.79)	0.001
N3	6.94 (3.36–14.33)	< 0.001	7.46 (3.31–16.83)	< 0.001
MLH1 positivity	2.03 (1.28–3.22)	0.003	2.10 (1.25–3.56)	0.005
Lauren classification				
Intestinal	1.00	1.000	1.00	1.000
Diffused	1.69 (0.68–4.20)	0.260	2.70 (0.97–7.50)	0.057
Mixed	1.75 (1.26–2.42)	< 0.001	1.63 (1.13–2.34)	0.009
Poor differentiation	1.60 (1.14–2.24)	0.006	1.51 (1.05–2.19)	0.028
ACRG subtype				
TP53 negative	1.00	1.000	1.00	1.000
TP53positive	0.78 (0.52–1.18)	0.246	0.82 (0.52–1.29)	0.391
MSI	0.52 (0.32–0.84)	0.008	0.48 (0.27–0.85)	0.012
EMT	1.56 (1.02–2.40)	0.041	1.62 (1.03–2.55)	0.037
Chemotherapy	0.48 (0.32–0.73)	< 0.001	0.49 (0.31–0.76)	0.001
Mesenchymal phenotype	1.79 (1.29–2.50)	< 0.001	1.92 (1.34–2.76)	< 0.001
9-gene score	1.29 (1.12–1.49)	< 0.001	1.31 (1.13–1.52)	< 0.001
MYOCD	1.34 (1.21–1.48)	< 0.001	1.37 (1.23–1.52)	< 0.001
GHRL	1.05 (0.97–1.14)	0.193	1.07 (0.98–1.16)	0.148
SCRG1	1.23 (1.15–1.32)	< 0.001	1.24 (1.15–1.34)	< 0.001
TYRP1	1.18 (1.08–1.30)	< 0.001	1.19 (1.08–1.31)	< 0.001
LYPD6B	1.02 (0.92–1.13)	0.760	0.99 (0.89–1.11)	0.896
THBS4	1.23 (1.13–1.34)	< 0.001	1.24 (1.14–1.36)	< 0.001
TNFRSF17	0.95 (0.87–1.04)	0.240	0.94 (0.86–1.04)	0.218
SERPINB2	0.98 (0.89–1.07)	0.653	0.94 (0.84–1.04)	0.225
NEBL	1.00 (0.92–1.09)	0.985	1.01 (0.92–1.11)	0.882
Borrmann type				
Borrmann I or EGC	1.00	1.000	1.00	1.000
Borrmann II	0.87 (0.37–2.08)	0.757	0.83 (0.34–1.99)	0.670
Borrmann III	1.70 (0.74–3.91)	0.209	1.42 (0.61–3.27)	0.417
Borrmann IV	4.07 (1.70–9.76)	0.002	3.33 (1.37–8.11)	0.008

**Table 6:** (A) Multivariate backward stepwise Cox regression on OS Training-Validation (N = 258, including 2 cases with NA entries). (B) Multivariate backward stepwise Cox regression on OS whole ACRG (N = 300, including 3 cases with NA entries)

	Hazard ratio	*P* value
(A) (N = 258)		
N stage		
0	1.00	1.000
1	2.77 (1.04–7.39)	0.042
2	2.06 (0.77–5.47)	0.149
3a	4.50 (1.75–11.56)	0.002
3b	8.78 (3.36–22.90)	< 0.001
Chemotherapy	0.48 (0.31–0.75)	0.001
SCRG1	1.21 (1.11–1.32)	< 0.001
(B) (N = 300)		
T stage		
T2-3	1.00	1.000
T4a	1.51 (1.03–2.20)	0.034
T4b	1.61 (0.91–2.85)	0.104
N stage (6th AJCC)		
N0	1.00	1.000
N1	1.71 (0.84–3.48)	0.143
N2	2.92 (1.42–6.00)	0.004
N3	5.14 (2.45–10.81)	0.000
MLH1 positivity	1.60 (1.00–2.57)	0.051
Chemotherapy	0.43 (0.28–0.66)	0.000
SCRG1	1.14 (1.05–1.24)	0.001

### Comparison of our signature with other gene signatures for stage prediction

**Fig. 7:**
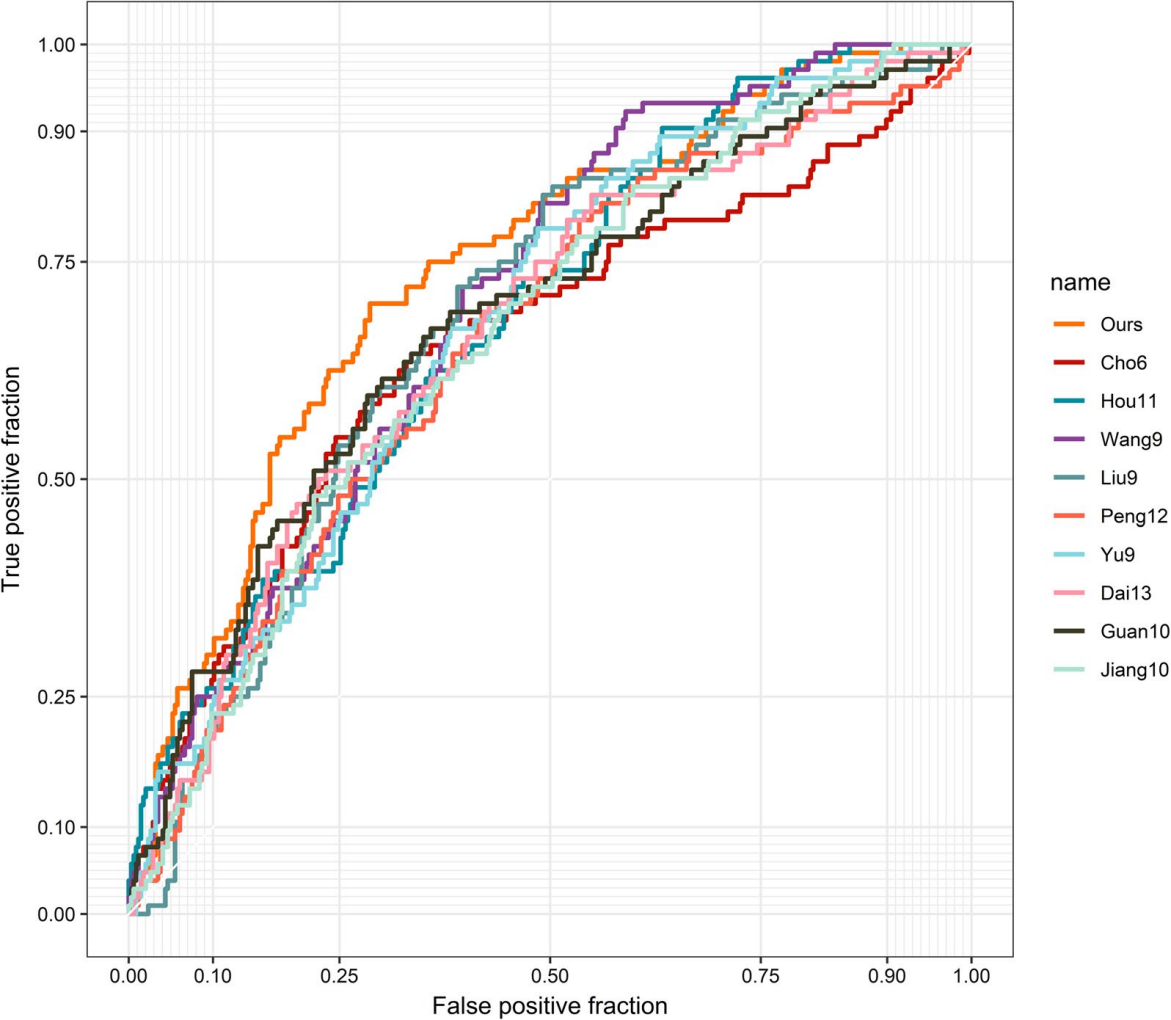
Receiver operating characteristic (ROC) curve analysis for stage prediction of our signature and other gene sets appeared in previous studies

A literature search was then performed, and the stage prediction ability of our signature was compared with those of nine other gene combinations containing similar gene numbers (ranging from 6 to 13 genes). The dataset for this analysis included the training-validation set and validation set 2 (N = 450) after batch correction. The coefficients were adjusted in all 10 signatures with the aim of achieving maximum predictive ability. Among the 10 gene collections, our signature achieved the highest AUC for stage prediction (AUC = 0.742, Fig. 7). The ROC curves indicated that our nine-gene signature was significantly different from the signatures reported in six studies and marginally significantly different from the signatures reported in three studies (Table 7).

**Table 7:** The collection of gene signatures of STAD used for comparison

Study	Genes	AUC	*P* value^†^
Ours	MYOCD, GHRL, SCRG1, TYRP1, LYPD6B, THBS4, TNFRSF17, SERPINB2, NEBL	0.742	1.000
Cho et al.	CTNNB1, EXOSC3, TOP2A, TRANK1, LZTR1, CCL5	0.657	0.003
Hou et al.	TRPC1, SGCE, TNFRSF11A, LRRN1, HLF, CYS1, PPP1R14A, NOVNBEA, CES1, RGN	0.686	0.026
Wang et al.	NR1I2, LGALSL, C1ORF198, CST2, LAMP5, FOXS1, CES1P1, MMP7, COL8A1	0.703	0.060
Liu et al.	TOP2A, COL1A1, COL1A2, NDC80, COL3A1, CDKN3, CEP55, TPX2, TIMP1	0.686	0.108
Peng et al.	ACOT7, CES1, IPMK, NES, PBX3, TMEM245, MIR6756, RAB11FIP4, RBPMS2, RPS27L, TPMT, TNFRSF11A	0.656	0.006
Yu et al.	MFAP2, SPP1, COL1A1, BGN, COL11A1, COL10A1, MXRA5, COMP, AGRN	0.681	0.046
Dai et al.	DCLK1, FLRT2, MCC, PRICKLE1, RIMS1, SLC25A15, SLCO2A1, CDO1, GHR, CD109, SELP, UPK1B, CD36	0.673	0.011
Guan et al.	HBB, C4orf48, MANEAL, CXCL3, TRIM31, TMEM200A, SERPINE1, F5, NOXO1, DKK1	0.685	0.101
Jiang et al.	AKAP12, ANGPTL1, CYS1, MLLT11, NAV3, NBEA, NOV, PTN, TUSC3, ZSCAN18	0.666	0.006

AUC: area under curve; ^†^*P*-value stands for Venkatraman permutation test

## Discussion

The present study identified 22 overlapping DEGs based on the integration of the TCGA and GEO public datasets. A nine-gene signature was formed based on LASSO regression results and was further validated in several sets with satisfactory AUC values of > 0.7 in most datasets. The moderate AUC performance in the GSE15459 dataset is likely due to the inconsistent grouping criteria used in this dataset; we were unable to deal with the stage migration problem due to a lack of clinical data. The significant improvement in the AUC after batch correction provides further verification of the stage distinguishing ability of our nine-gene signature. The nine-gene signature reported here is the first stage-oriented prediction model at the transcriptome level using the AJCC 8th edition TNM staging system. The results suggest that this nine-gene signature may be of diagnostic value for the management of non-metastatic STAD and may assist with clinical decision-making.

For historical reasons, most current open-access gene expression sets for STAD have followed the AJCC 6th edition stage classification. The well-known “GEPIA” tool, for example, integrated various datasets and finalized a “stage plot” module [28]. Despite this excellent work and contribution to the field, this approach is somewhat open to question because, from the viewpoint of gastroenterologists and clinical oncologists, the relationship between the 6th staging system and the newest 8th TNM staging system is by no means a simple permutation or combination. For example, the AJCC 6th edition categorizes muscularis propria invasion as pT2a, subserosal invasion as pT2b, serosal penetration as pT3, and adjacent organ invasion as pT4, which corresponds to pT2, pT3, pT4a, and pT4b T stage criteria in the 8th (and 7th ) editions [29, 30]. Even more importantly, both the 5th and 6th editions defined N1, N2, and N3 as positive lymph node numbers of 1–6, 7–15, and > 15, respectively, while starting from the 7th edition, the N stages were further refined as N1: 1–2, N2: 3–6, N3a:7–15, and N3b: > 15 positive lymph nodes. This means that there is a considerable discrepancy when discussing the association between gene expression/behavior and stage [28]: patients with the same “N2” staging according to the 6th and 8th editions reflect different concepts and prognoses which cannot be simply merged together [9, 31]. Additionally, stage migration is another key factor in translating stages from the old to the new system and is a precondition for explaining the expression differences between earlier- and later-stage samples (Additional file 1: Table S1). Our previous findings of 1663 patients indicated that prognosis differences begin to reach statistical significance when pTNM stage reaches IIB [32]. This result prompted us to split the data in the current study into earlier- and later-stage patient groups as these groups are associated with prognosis and treatment strategy variations. Finally, as retrieval of > 15 lymph nodes is required for optimal staging, samples with inadequate lymph node retrieval are at considerable risk of under-staging and should be filtered out in analyses [33–36]. Given the above, due to the strict data processing performed in this study, these data can be used for accurate stage prediction and to identify factors (DEGs and pathways) that lead to stepwise STAD progression.

Based on TNM stage characteristics, 22 overlapping DEGs were identified between the TCGA set and the training set. This number is similar to that in a prognosis-based study [37], but is far less than those obtained in other data mining studies that have focused on gene expression between tumor and normal tissue. This suggests that either the homogeneity or heterogeneity among gastric adenocarcinomas is much more complex. Accordingly, a penalized model (LASSO regression) was implemented to exclude the confounding variables that could generate multicollinearity in the prediction model. In fact, the coefficients of the nine selected genes in the multivariate analysis maintained the same sign as in the univariate models. This confirms the robust performance of the model. This model also avoided overfitting and the Simpson’s Paradox, which are risks when performing bioinformatics analysis and model building [37–40]. More importantly, our signature had higher accuracy for stage prediction than previous signatures focusing on various prognostic features. Therefore, the results of this study indicate that the nine-stage signature is a novel biomarker with superior tumor stage predictive ability for LAGC patients.

Of the nine identified genes, some have been reported to be of relevance to various cancers. THBS4 is one of five extracellular calcium-binding proteins that modulate the extracellular matrix (ECM). High levels of THBS4 have been found to be significantly related to cancer-associated ECM in breast cancer tissue [41], and the high expression levels of THBS4 in cancer-associated fibroblasts in Lauren diffuse-type gastric adenocarcinoma support its use as a biomarker [42]. Clinically, the Lauren type has been shown to be strongly correlated with lymph node metastasis in STAD [43]. In vitro, THBS4 also promotes tumor progression by interacting with ITGB1 via the FAK/PI3K/AKT pathway [44, 45].

Tyrosinase-related protein 1 (TYRP1) is the most abundant intracellular glycoprotein in melanoma and melanocytes [46]. Although it has a specific function in melanogenesis, it seems that high expression profiles of TYRP1 are not exclusive to melanoma. Bioinformatics analyses have demonstrated similar unusual overexpression of TYRP1 in STAD, and its expression is associated with poorer prognosis [8, 47]. It is proposed that the high expression of TYRP1 could serve as an indicator of the abnormal activation of transcription regulator microphthalmia-associated transcription factor (MITF), which is phosphorylated by the SCF/KIT pathway, or of the inactivation of anti-oncogenes like p53, which results in tumor progression [48–50]. Furthermore, TYRP1 mRNA has been proven to cause ncRNAs to function as sponges for miR-16, which is known for its tumor-suppressor function in STAD [51, 52]. All of the above evidence indicates that TYRP1 plays a role in STAD progression.

SERPINB2, commonly known as plasminogen activator inhibitor-2 (PAI-2), serves as an inhibitor of extracellular protease urokinase plasminogen activator (uPA) and tissue plasminogen activator (tPA), both of which transform plasminogen into plasmin [53]. uPA-triggered fibrinolysis plays various roles in tumor progression, including ECM degradation, the release of tumor-related growth factors, and the promotion of angiogenesis [54–56]. In vitro, SERPINB2-deficient cancer cells are associated with increased tumor growth, aberrant ECM, and invasive properties, while SERPINB2 overexpression inhibits tumor proliferation and migration [57, 58]. A low-expression profile of SERPINB2 is linked with poor prognosis in various cancers, including STAD [7, 59].

The GHRL gene encodes the prepropeptides of ghrelin and obestatin. Physiologically, ghrelin/obestatin stimulate/decrease food intake, regulate growth hormones, and may have a role in cell proliferation, differentiation, and apoptosis [60, 61]. In vitro, ghrelin is reported to induce colon cancer cell proliferation through the GHS-R/Ras/PI3K/Akt/mTOR axis [62]. Abnormally high expression of GHRL is not only observed in gastrointestinal tumors but also in other types of cancer including breast cancer, renal cell carcinoma, and ovarian cancer [63, 64]. Interestingly, although in vitro studies and expression arrays have suggested stimulatory effects of ghrelin on proliferation and invasion of STAD, several clinical studies have indicated that ghrelin in serum acts as a protective factor for STAD patient prognosis [65, 66]. This suggests that circulating ghrelin and tumor-localized ghrelin have different effects [67]. A more comprehensive mechanistic analysis is needed to explain this phenomenon.

Scrapie responsive gene 1 (SCRG1) is predominantly expressed in neurons and is overexpressed in the central nervous system during infection or brain injury [68]. SCRG1 was initially recognized as a marker of autophagic vacuoles in terminal-stage disease [69]. The upregulation of SCRG1 was previously reported in STAD with lymph node metastasis in a data-mining study; however, the mechanism was not explained [70]. More recent studies have revealed that SCRG1 acts on CD157 to activate ERK and PI3K/Akt in human mesenchymal stem cells [71, 72]. SCRG1 is also specifically highly expressed in breast cancer with metastatic propensity [73] and might serve as an ideal indicator for developmental cancer-associated fibroblasts [74].

NEBL is also a commonly distinguishable gene that serves as a prognostic factor in various cancers, according to previous microarray results [75, 76]. Because the nebulette protein encoded by the NEBL gene mostly functions to stabilize actin filaments, the expression level of NEBL may reflect the extent of focal adhesion of anchored cancer cells [77]. Contrary to previous findings in colorectal cancer, whereby Hosseini et al. discovered a positive correlation between the expression level of NEBL and lymph node metastasis, the bioinformatics-based analysis in the present study revealed a negative correlation between these two factors. It is proposed that a stabilized cytoskeletal structure results in less random motility, thus enhancing focal adhesion and predicting late-stage STAD with poorer prognosis [78, 79].

Among the remaining three genes, the tumor necrosis factor receptor superfamily member 17 (TNFRSF17) gene, also known as the B-cell maturation antigen gene, is expressed on mature B cells and directly reflects B-cell homeostasis and autoimmune response [80]. The expression of TNFRSF17 is associated with the development of breast cancer, ovarian cancer, and colon cancer [81–83]. TNFRSF17 also has the potential to act as a marker for evaluating tumor immune infiltration status and it may predict beneficial effects of immune checkpoint blockade antigens [84–86]. Interestingly, in the current study, although TNFRSF17 showed a higher expression profile in later-stage samples, it had no effect on patient survival. In fact, the role of B cells in tumorigenesis and progression is much less understood than other immune cells [87, 88]. This may be due to the two-pronged nature of B cells [87]. On the other hand, the relationship between the overexpression of TNFRSF17 and its global contribution to/reflection of the tumor microenvironment requires further study [89]. LYPD7, also known as LYPD6B, belongs to the LY6/PLAUR domain-containing subclass (LYPD) of the Ly-6/uPAR superfamily [90]. Several bioinformatics-based analyses have revealed that increased LYPD7 expression may be implicated in the pathogenesis of NSCLC, while decreased hypermethylation of LYPD DNA is correlated with an invasive phenotype of malignant melanoma [91, 92]. Finally, contrary to the MYOCD profile in other common tumors, in which myocardin plays a suppressive role in the malignant transformation process [93, 94], the MYOCD level in STAD was vastly upregulated, indicating poorer prognosis (Additional file 8: Figure S4). This MYOCD amplification should be comprehensively investigated because activation of the PI3K/Akt pathway can lead to JAK3 phosphorylation, thus resulting in a STAT3 and myocardin interaction which co-regulates smooth muscle cell proliferation and angiogenesis [95].

Given that the limited number of DEGs identified in this study may not provide a robust enrichment analysis, GSEA was used to inspect the pathways involved in STAD development, with samples grouped by stage. GSEA analysis revealed that the PI3K-Akt, MAPK, and calcium signaling pathways are the top three pathways correlated with later-stage STAD compared to earlier-stage STAD. All three pathways play a vital role in cell proliferation, growth, and apoptosis escape, which are indicative of the higher proliferative profile of late-stage STAD. Based on the network analysis, the proliferation-related and metabolic-related pathways are two major modules that are widely upregulated in stage advancement, while immune-related and DNA repair-related genes are widely downregulated. These results suggest that the development and migration of STAD depend on the stepwise activation of these commonly dysregulated pathways in cancer. Additionally, the GSEA analysis provides solid evidence of changes in tumor behavior according to tumor stage.

As most genes identified in this study were linked with the genesis and development of STAD, the increase in the nine-gene score resulted in a poorer prognosis. Among the nine identified genes, MYOCD, SCRG1, TYRP1, and THBS1 were statistically associated with patient survival, while GHRL, LYPD6B, SERPINB2, and NEBL only showed trends toward better or worse prognosis. Using stepwise backward elimination, only SCRG1 was an independent prognostic factor. This result is understandable because the stepwise algorithm is designed to mathematically avoid multicollinearity [96, 97]. This method is advantageous when the significance of covariates is unknown and the covariates are equally weighted [98]. Since our nine-gene signature was designed to predict tumor stage, a higher correlation with the T or N stage is unavoidable (Table 3), and several stage-related genes can be ruled out when the N and T stages become two of the most important prognostic factors. Apart from T and N stage, chemotherapy and MLH1 status are two clinicopathological features that significantly influence OS. Other important features, including the Lauren classification, ACRG subtype, and mesenchymal phenotype were also excluded from the Cox model due to multicollinearity. To read beyond the analysis, we hypothesize that the results shed light on a simple idea that some genomic or transcriptomic results might be products of an overfitting model using a limited sample size. Nonetheless, a population-based transcriptomic result is still necessary. Meanwhile, several key clinical features (e.g., chemotherapy management) and phenotypes (e.g., TNM stage) are still key factors that drive patient prognosis. Moreover, as several key clinical features are successively related, it is important to focus on the correlation between transcriptomic signatures and key cancer phenotypes to prescribe individualized treatment for patients. Based on this, the nine-gene signature identified in this study can assist with accurate STAD staging.

Clinically, our stage-related gene signature could support decision-making in several ways. First, as preoperative diagnosis has become increasingly important in the multimodality treatment of patients who are initially diagnosed with locally-advanced gastric cancer, a chip-based panel facilitates accurate clinical staging where diagnostic accuracy to date has been limited by the use of enhanced CT [99, 100]. Patients who are over-staged could receive timely resection, while under-staged patients may benefit from systemic treatment before surgery. Second, for D0/D1 surgery or D2 with limited lymph node retrieval number (< 15), a stage-related gene panel allows for tumor restage and more accurate forecasting of the risk of lymph node metastasis, which can inform clinicians’ postoperative regimen choices. Third, for early gastric cancer (T1a/1b) with endoscopic resection, the signature identified here can be used to help decide whether salvage surgery is needed, as it is highly linked with lymph node metastasis and infiltration [101, 102]. Similarly, an extended lymphadenectomy or extensive radical resection may improve long-term outcomes for patients with staggeringly high signature scores [103, 104]. To sum up, more precise preoperative staging can be achieved collaboratively using radiological and transcriptomic methods.

This study has some limitations that should be noted. First, the prediction model was based on bioinformatic analysis and lacked its own validation cohort. Second, although a stringent data washing workflow was implemented, there were still some under-staged samples due to missing information in the public dataset. Third, the nine-gene signature is a probe-based model limited to the GPL570 platform; cross-platform validation may require systemic correction considering the different sensitivity of gene probes in each platform. Fourth, although the nine-gene signature exhibited promising predictive ability, the present model was mRNA-based. The performance of our signature should be further explained by the regulation of corresponding non-coding RNA, otherwise the consistency of associations across genomic and protein-level needs further inquiry. Finally, yet importantly, both GSEA and conventional enrichment analysis methods were used to investigate the expression profile differences between groups, but results that drawn from each method were fragmented. According to GSEA, the Calcium and MAPK signaling pathways achieved high normalized enrichment scores. However, none of the nine genes were involved in these two pathways. It is obvious that biological meanings were limited by the gene number of mathematical optimum, which need our further expansion.

## Conclusion

In summary, under stringent data filtering, nine hub genes were identified. These genes predict stage advancement in gastric adenocarcinoma. This nine-gene signature may help facilitate clinical decision-making for patients with localized STAD of uncertain stage. This model may also assist with tumor staging/restaging, especially for those patients with insufficient lymph node retrieval. Nevertheless, further analysis of the molecular mechanisms underlying the roles of these hub genes is required, as well as identification of the factors that drive activation/deactivation of the pathways involved in STAD progression.

## Supplementary Information

**Additional file 1: Table S1.** Stage variation in general view.

**Additional file 2: Table S2.** Gene expression set and clinical information of valid cases with clear stage and lymph sufficient lymph nodes retrieval in TCGA (n = 159) and training-validation (GSE62254, n = 262) before outliers removal; And test set2 (GSE15459, n = 192) with expression and clinical information.

**Additional file 3: Table S3.** Detailed information of the 22 overlapped DEGs.

**Additional file 4: Table S4.** The details of the significantly enriched gene sets using GSEA analysis.

**Additional file 5: Figure S1.** Outliers detection in (A) training-validation set (GSE62254) and (B) TCGA dataset.

**Additional file 6: Figure S2.** The Kaplan‑Meier curve of the nine hub genes in predict patients overall survival.

**Additional file 7: Figure S3.** The box plot of expression data (A) before and (B) after the batch effect correction for the GSE15459. The training-validation expression set was used as reference.

**Additional file 8: Figure S4.** The overexpression of MYOCD indicated better overall survival in (A) breast cancer, (B) ovarian cancer and (C) lung cancer. Contracting survival outcomes can be observed in (D) gastric cancer. Data from KM-plotter.

## Data Availability

The datasets analyzed during the current study are available in TCGA (https://portal.gdc.cancer.gov/), and GEO (https://www.ncbi.nlm.nih.gov/geo/query/acc.cgi?acc=GSE62254; https://www.ncbi.nlm.nih.gov/geo/query/acc.cgi?acc=GSE15459). The authors ensure the availability of supporting data and materials which can be obtained from the supplementary materials.
