# Supplementary material for: A 9‑gene expression signature to predict stage development in resectable stomach adenocarcinoma
Source: BMC Gastroenterol. 2022 Oct 14;22:435. doi: 10.1186/s12876-022-02510-8 (PMC9564244; doi:10.1186/s12876-022-02510-8)
Supplement: Supplementary file 1 — Additional file 1: Table S1. Stage variation in general view. [file 12876_2022_2510_MOESM1_ESM.docx]

Table S1. Stage migration in general view

| AJCC 8^th^ | N0 | N1 (1-2) | N2 (3-6) | N3a (7-15) | N3b (>15) |  |
| --- | --- | --- | --- | --- | --- | --- |
| T1 | IA  IA | IB  IB | IIA  IB | IIB  II | IIIB  IV | T1 |
| T2 | IB  IB | IIA  II | IIB  II | IIIA  IIIB | IIIB  IV | T2a |
| T3 | IIA  IB | IIB  II | IIIA  II | IIIB  IIIB | IIIC  IV | T2b |
| T4a | IIB  II | IIIA  IIIA | IIIA  IIIA | IIIB  IIIB | IIIC  IV | T3 |
| T4b | IIIA  IIIA | IIIB  IV | IIIB  IV | IIIC  IV | IIIC  IV | T4 |
|  | N0 | N1 (1-6) | N1 (1-6) | N2 (7-15) | N3 (>15) | AJCC 6^th^/5^th^ |
